# Modeling and solving staff scheduling with partial weighted maxSAT

**DOI:** 10.1007/s10479-017-2693-y

**Published:** 2017-11-07

**Authors:** Emir Demirović, Nysret Musliu, Felix Winter

**Affiliations:** Database and Artificial Intelligence Group, Vienna University of Technology, Vienna, Austria

**Keywords:** Employee scheduling, maxSAT, SAT encodings, Cardinality constraints

## Abstract

Employee scheduling is a well known problem that appears in a wide range of different areas including health care, air lines, transportation services, and basically any organization that has to deal with workforces. In this paper we model a collection of challenging staff scheduling instances as a weighted partial Boolean maximum satisfiability (maxSAT) problem. Using our formulation we conduct a comparison of four different cardinality constraint encodings and analyze their applicability on this problem. Additionally, we measure the performance of two leading solvers from the maxSAT evaluation 2015 in a series of benchmark experiments and compare their results to state of the art solutions. In the process we also generate a number of challenging maxSAT instances that are publicly available and can be used as benchmarks for the development and verification of modern SAT solvers.

## Introduction

Staff scheduling problems arise whenever there is the need for efficient management and distribution of workforce over periods of time. Therefore, a wide range of different institutions can benefit from an optimized staff schedule, including hospitals, airlines, security personnel, transportation services, and basically any organization that has to deal with a large number of employees. Finding the ideal workforce roster however is not an easy task, and even a basic variant of this problem belongs to the class of NP-hard problems (Chuin Lau 1996).

A variety of different staff scheduling problems are described in the literature and many solving methods have been proposed in the past. Corresponding surveys regarding employee scheduling were provided by Ernst et al. (2004) and Bergh et al. (2013). While many approaches are based on mathematical programming and heuristic methods, the application of solution strategies which model the problem as a maximum Boolean satisfiability problem (maxSAT) has not been considered. The intuitive way of working with propositional formulas, as well as growing developments in the SAT community motivate the investigation of such an approach.

In this paper we concentrate on the employee scheduling instances introduced by Curtois and Qu (2014). According to the authors those instances were designed to describe realistic and challenging staff scheduling problems while still being straightforward to use. The included scheduling periods range from one week up to one year, requiring up to 180 employees and 32 shift types to be assigned.

Recent publications provided an integer programming (IP) model as well as a metaheuristic approach to this problem. The best known solutions using these techniques as well as a description of the IP formulation were provided by Curtois and Qu (2014). A detailed description of the used algorithms, namely a branch and price method and a metaheuristic based on ejection chains can be found in the literature (Burke and Curtois 2014; Burke et al. 2008). With the use of the branch and price algorithm and ejection chains, optimal solutions could be found for most of the smaller instances and new lower/upper bounds could be determined for many instances. However, optimal solutions for a number of instances are still unknown.

In this paper we want to investigate a new solving paradigm for this problem based on maxSAT. Modeling a problem with a maximum propositional satisfiability formulation has shown to perform well on a variety of different applications in the past, including the scheduling of business-to-business meetings (Bofill et al. 2015) and High School Timetabling (Demirovic and Musliu 2014). The most related papers to our work are Kundu and Acharyya (2008) and Haspeslagh et al. (2013). Kundu and Acharyya (2008) proposed a SAT formulation for a basic nurse scheduling problem. The local search algorithm GSAT was used to provide solutions for the generated SAT instances. Haspeslagh et al. (2013) investigated the translation of instances from the first international nurse rostering competition (Haspeslagh et al. 2014) to SAT. Additionally, in this work the features of SAT instances were used to analyze the hardness of nurse rostering instances. To the best of our knowledge we investigate for the first time the application of maxSAT for employee scheduling. We also experiment with different encodings for the cardinality constraints and evaluate complete maxSAT solvers for the considered instances. Additionally, to show that the applicability of maxSAT is not restricted to the instances from Curtois and Qu (2014), we will describe how the maxSAT formulation introduced in this paper can be adapted to model the well known problem instances from the second international nurse rostering competition (INRC2) (Ceschia et al. 2015). Although several approaches have been applied on those instances within the competition (e.g. Römer and Mellouli 2016), to the best of our knowledge they will be modeled for the first time using maxSAT.

The main contributions of this paper are:

- We provide the first maxSAT formulation for the variant of the employee scheduling problem introduced by Curtois and Qu (2014). Furthermore, we show how the proposed maxSAT formulation can be adapted to model another well known variant from nurse rostering.
- We experiment with different encodings for cardinality constraints and compare two leading solvers from the maxSAT evaluation 2015. Additionally, we experiment with a simplification of the problem and provide a comparison with the state of the art solutions.
- We provide challenging instances which can be used by the maxSAT community to test and improve results of maxSAT solvers.

In Sect. 2 we first give a problem description to provide a deeper insight into the problem. We provide a brief introduction into the maximum satisfiability problem (maxSAT) and give the details of our maxSAT model in Sect. 3. In Sect. 4 we then present our experimental environment as well as the experiments which have been conducted. At the end of the paper in Sect. 5 we make final conclusions and provide an outlook on future work.

## Problem description

In our work we deal with a variant of the employee scheduling problem as it was described by Curtois and Qu (2014). We chose to focus on this specific problem formulation as it provides a number of instances that include challenging and realistic scheduling problems, while still being intuitive and straightforward to use.

The overall goal is to find an optimal roster for a number of given employees and shift types, where every employee may either work a single shift or have a day off on each day of a given scheduling period. For this problem the scheduling period is stated as a number of weeks and therefore the number of days is always a multitude of seven. Another property concerning the scheduling horizon ensures that the first day of the roster is always a Monday, while the last day is always a Sunday. The employees and shift types which are considered in this problem are specified by a list of unique names which are connected with a number of constraints that restrict all possible shift assignments. Some employees might for example be only allowed to work in certain shifts and patterns of consecutive working shifts might be prohibited or requested. Each problem instance specifies hard- and soft-constraints to set up a corresponding rule set. Hard constraints on the one hand are always strict and have to be fulfilled in order to generate a feasible solution. Soft constraints on the other hand may be violated, but will in case of a violation lead to an integer valued penalty. For example a hard constraint in our problem could restrict the minimum and maximum amount of time that an employee has to work in total over the whole scheduling horizon. Personal shift requests that employees can state are formulated as soft constraints in our problem instances.

Finally, the objective function of a candidate solution is defined as the sum of all violated soft constraints. We therefore deal with an optimization problem, where the optimal solution is the schedule with the lowest possible objective value. We have a deeper look on all of the constraints in the next section.

## Modeling employee scheduling as partial weighted maxSAT

### The maximum satisfiability problem

The satisfiability problem (SAT) is a decision problem which asks whether there exist assignments of truth values to variables, such that a propositional logic formula is evaluated true (that is, the formula is satisfied). A propositional logic formula is built from Boolean variables using logic operators and parentheses. The formula is usually given in conjunctive normal form (CNF), meaning that the formula is a conjunction of clauses, where a clause is a disjunction of literals, where a literal is a variable or its negation. For example, the formula $(X_1 \vee X_2) \wedge (\lnot X_1 \vee \lnot X_3)$ is said to be satisfiable, because the assignment $(X_1, X_2, X_3) = (true, false, false)$ satisfies the formula. However, had we inserted the clause $(\lnot X_1 \vee X_2 \vee X_3)$, the same assignment would no longer satisfy the formula.

An extension to SAT that we consider in this work is Partial Weighted maxSAT, in which clauses are partitioned into two types: hard and soft clauses. Each soft clause is given a weight. The goal is to find an assignment which satisfies the hard clauses and minimizes the sum of weights of the unsatisfied soft clauses. For more in depth information about SAT and maxSAT, we direct the interested reader to the literature (Biere et al. 2009).

In the following sections we will formulate our problems as maxSAT. The obtained maxSAT formulas which model the problem are called encodings.

### Decision variables

In order to model the assignment of shifts to employees, we define variables $S_{i,d,t}, \forall i \in I, d \in D, t \in T$, where *I* denotes the set of all employees, *D* refers to the set of days in the planning horizon, and *T* is the set of all shift types in the problem. Each variable $S_{i,d,t}$ will be set to true if and only if employee *i* gets the shift type *t* assigned on the *d*-th day in the roster, otherwise it will be set to false. Additionally, we define helper variables $X_{i,d}, \forall i \in I, d \in D$ which are set to true if employee *i* has no shift assigned on day *d*. So $X_{i,d}$ is set to true if and only if employee *i* is considered to have a day off on this day.

To connect the *X* variables with the decision variables *S* we include the following equivalences in our formulation:

1 $$\begin{aligned} X_{i,d} \leftrightarrow \bigwedge \limits _{t \in T} \lnot S_{i,d,t} \quad \forall i \in I, d \in D \end{aligned}$$

In the following sections we give a description of all the constraints in our employee scheduling problem and additionally specify how each of them is encoded in our partial weighted maxSAT formulation. Clauses which are generated from soft constraints will also have weights assigned.

Since many of the constraints contain properties of cardinality constraints, we continue by shortly introducing the notion of them.

### Cardinality constraints

In order to be able to formulate all of the constraints for the problem, it is necessary to make use of cardinality constraints. Such constraints define limits on the number of truth assignments on a set of given Boolean variables. There are three different types of cardinality constraints: $\textit{atLeast}_k(x_i : x_i \in X)$, $\textit{exactly}_k(x_i : x_i \in X)$, and $\textit{atMost}_k(x_i : x_i \in X)$ which are defined on sets of variables that should have at least, exactly, or at most *k* variables having their truth value assigned. For example if a cardinality constraint limits the number of true valued variables of the set ${x_1,x_2,x_3}$ to at most two $\textit{atMost}_2(\{x_1,x_2,x_3\})$, the assignment $(x_1,x_2,x_3) = (1,1,0)$ is considered to be feasible, while the assignment $(x_1,x_2,x_3) = (1,1,1)$ would be considered as infeasible.

Additionally, we have to distinguish between hard- and soft cardinality constraints. While hard cardinality constraints decide whether or not the overall solution will become feasible, soft cardinality constraints will only penalize the objective function if violated. In our problem we assign a weight to a cardinality constraint and calculate the total penalty linearly depending on the difference to the violated limit. For example if we consider the constraint $\textit{atLeast}_2(\{x_1,x_2,x_3\})$ with a weight of 40, the assignment $(x_1,x_2,x_3) = (0,0,0)$ would lead to a penalty of $40 \cdot 2 = 80$.

Different variants of dealing with cardinality constraints in Boolean satisfiability problems have been studied in the literature (e.g. Sinz 2005; Asín et al. 2009). In this paper we investigate four different encoding types: combinatorial encoding, sequential encoding, bit adder encoding, and cardinality networks. The encodings differ in the number of variables and clauses needed, and whether or not *unit propagation* is enough to ensure *generalized arc consistency*. Unit propagation forces the assignments of variables that are contained in *unit clauses*, that is, clauses which consist of only a single literal. Since all clauses must be satisfied, including unit clauses, the value of a variable must be set such that the unit clause is satisfied. A constraint is generalized arc consistent if for each unassigned variable and value that it can be assigned, there exists an assignment of values to the other unassigned variables in the constraint such that the constraint is satisfied (Mohr and Masini 1988). It is important to note that in our modeling, whether an employee scheduling constraint is generalized arc consistent or not solely depends on generalized arc consistency of the cardinality constraint used. In the following we briefly describe the cardinality constraints used, we refer the reader to Demirovic and Musliu (2014), Sinz (2005), and Asín et al. (2009) for more details.

#### Combinatorial encoding

The combinatorial encoding enumerates all possible undesired truth assignments and forbids them explicitly by generating corresponding clauses. While this approach may provide an efficient encoding for small cardinality constraints, the number of generated clauses will grow exponentially with the number of variables. An alternative approach would be to explicitly enumerate all desired truth assignments. For example, to encode $\textit{atLeast}\_2(\{x_1, x_2, x_3\})$, one needs to encode clauses that forbid every assignment which does not have at least two variables $x_1$, $x_2$, and $x_3$ set to true. This can be achieved with the following clauses: $(x_1 \vee x_2 \vee x_3)$, $(x_1 \vee x_2 \vee \lnot x_3)$, $(x_1 \vee \lnot x_2 \vee x_3)$, and $(\lnot x_1 \vee x_2 \vee x_3)$. The first clause requires that at least one variable is set to true, while the other three forbid assignments in which exactly one variable is set to true, encoding the desired constraint. Unit propagation ensures generalized arc consistency.

#### Sequential and bit-adder encoding

The main idea behind the sequential and bit adder encoding is to encode the sum of the considered variables $x_i$ and then forbid certain output values. However, this cannot be done directly in SAT. Thus, one must introduce auxiliary variables and clauses to capture the desired behavior.

Both encodings use additional variables to represent each partial sum of the original variables. The *i*-th partial sum is defined in terms of the *i-th* variable and the ${(i-1)}$-th partial sum ($ps(i) = ps(i-1) + x_i$ and $ps(0) = 0$). Afterwards, a constraint on the last partial sum is imposed to encode the cardinality constraint. For example, for the assignment $(x_1, x_2, x_3, x_4) = (1, 0, 1, 1)$, $ps(1) = 1$, $ps(2) = 1$, $ps(3) = 2$, and $ps(4) = 3$. Enforcing $ps(4) = 3$ would lead to the $\textit{exactly}\_3$ cardinality constraint.

In SAT, variables (e.g. for *ps* above) cannot be assigned numeric values. Therefore, they need to be encoded with Boolean variables instead. This is main difference between the sequential and bit-adder encoding: the former uses unary, while the later uses binary number representations.

A number $u \in [0, n]$ can be represented as unary in SAT with *n* additional variables $u_i$ and the following constraints defined on them:

2 $$\begin{aligned} \bigwedge _{i \in [1, n-1]} (u_i \Rightarrow u_{i-1}). \end{aligned}$$

For example, for the variable $u \in [0, 5]$, five auxiliary variables are introduced and the assignment $u = 3$ would be given by $(u_1, u_2, u_3, u_4, u_5) = (1, 1, 1, 0, 0)$. The addition operation between can be defined on unary number with additional clauses and variables. The same number may be represented in binary with *log*(*n*) $b_i$ variables: $(b_2, b_1, b_0) = (1, 0, 1)$.

Due to the different number representations, the bit-adder encoding requires less clauses and variables when compared to the sequential encoding (*O*(*nlog*(*n*)) clauses and variables as opposed to $O(nk - k^2)$ variables and *O*(*kn*) clauses), but unit propagation is not sufficient to ensure generalized arc consistency for the bit-adder, while it is for the sequential encoding. For more details on the encodings, we refer the reader to Demirovic and Musliu (2014), Sinz (2005), and Asín et al. (2009).

#### Cardinality networks

Cardinality networks generate helper variables that are used to sort all the considered truth assignments and then insert clauses which forbid certain outputs. The sorting is performed in a similar way as a simple merge sort algorithm would work. For example, considering an assignment $(x_1 = 0, x_2 = 1, x_3=0, x_4=1)$, the helper variables $a_{1-4}$ would represented the sorted version of this assignment $(a_1 = 1, a_2 = 1, a_3 = 0, a_4 =0)$. Additional clauses are then inserted to forbid undesired assignments of the helper variables. Generalized arc consistency is maintained by unit propagation. The encoding requires $O(nlog^2k)$ auxiliary variables and clauses. More details can be found in Asín et al. (2009).

In the experiments covered in later sections of this paper we compare the performance of those four encodings on two maxSAT solvers.

### Modeling of hard constraints

In this section we give a description of the hard constraints for employee scheduling problem which is considered in this paper and specify how these constraints can be encoded in our partial weighted maxSAT formulation.

*An employee cannot be assigned more than one shift on a single day* Since no employee should work two shifts on the same day, we have to ensure that no two variables $S_{i,d,t}$ and $S_{i,d,x}$ may be set to true at the same time if $t \ne x$ and $i \in I$, $d \in D$, $t,x \in T$ where *I* is the set of all employees, *D* is the set of all days in the scheduling horizon and *T* is the set of all possible shift types.

We model this constraint with an $\textit{atMost}_1$ cardinality constraint.

3 $$\begin{aligned} \textit{atMost}_1(\{S_{i,d,1},S_{i,d,2},\dots ,S_{i,d,|T|}\}) \quad \forall i \in I, d \in D \end{aligned}$$

*Disallowed shift sequences* It is required that each employee needs to rest for a minimum amount of time after he has worked in a shift. The length of the necessary rest period varies for each shift type. Because each shift has fixed starting and ending times during the day, the set of shift types that cannot follow a certain shift type *t* can be determined easily by considering all pairs of shift types and comparing their difference in start and ending times with the minimum rest time. We refer to the set of all shift types that are not allowed to follow a shift *t* as $R_t$.

The constraints can therefore also be thought of as a number of disallowed shift sequences which consist of two consecutive shifts and can be included in our formulation by inserting a clause for each sequence.

4 $$\begin{aligned} \bigwedge \limits _{d=1}^{|D|-1}( S_{i,d,t} \rightarrow \lnot S_{i,d+1,x}) \quad \forall t \in T, x \in R_t \end{aligned}$$

*The maximum numbers of shifts for each type that can be assigned to an employee* In our problem some of the employees can have contracts which only allow them to work in specific shift types for a maximum number of days. For example such a limit could restrict the number of night shifts an employee may work during the schedule to four, making any roster which assigns five night shifts to a single employee infeasible. The maximum numbers for each employee and shift type are given as parameters $m_{it}^{max}$ with the problem instances, where $i \in I$ and $t \in T$.

Since this constraint can be seen as the basic case for a cardinality constraint, we do not discuss the detailed encoding into Boolean satisfiability clauses here, but simply state it as an $\textit{atMost}$ cardinality constraint instead:

5 $$\begin{aligned} \textit{atMost}_{m_{it}^{max}}\left( \{S_{i,1,t},S_{i,2,t},\dots ,S_{i,|D|,t}\}\right) \quad \forall i \in I, t \in T \end{aligned}$$

*Minimum and maximum working time* Each shift type assigns a certain amount of working time in minutes to its associated employees. Moreover the total amount of the working time in minutes is restricted for each employee and must lie between a minimum and maximum bound. Those limits are given to the problem in form of the parameters $b_i^{min}$ and $b_i^{max}$ for each $i \in I$.

In order to formulate this constraint efficiently, we introduce additional helper variables which help us to count the total number of minutes worked by an employee. For their definition we consider the shift lengths $l_t, \forall t \in T$ which are given as parameters to the problem and specify the number of working time in minutes required for shift *t*. Furthermore we define their greatest common divisor $g = gcd(l_t : t \in T)$. If we have three different shift types, with the first one lasting for 480, the second one lasting 620, and the third one lasting 120 min, *g* would then be 20 for example.

With *g*, we can then calculate simplified lengths for all shifts $$sl_t = {l_t \over g}$$$$\forall t \in T$$. Additionally, we define the maximum simplified shift length $$sl_{max} = max\{sl_t : t \in T\}$$. Now we are able to introduce our variable set *U*, which counts the units of time an employee *i* works on day *d*.

For each employee and day, we introduce helper variables $U_{i,d,x}, \forall i \in I, d \in D, x \in {1,\dots ,sl_{max}}$ and set up a number of equivalences in order to correctly connect them with our decision variables *S*:

6 $$\begin{aligned} S_{i,d,t} \leftrightarrow \bigwedge \limits _{x=1}^{sl_t} U_{i,d,x} , \bigwedge \limits _{y=sl_t}^{sl_{max}} \lnot U_{i,d,y} \end{aligned}$$

All of the *U* variables can now be used to count the overall units of time an employee works and we can use them in order to set up two cardinality constraints that ensure the minimum and maximum working time constraint. Note that since we are using simplified lengths, we also have to divide the given limits by the common divisor *g* and round appropriately:

7 $$\begin{aligned} \textit{atMost}_{\lfloor b_i^{max}/g \rfloor }\left( \{U_{i,d,x} | d \in D, x \in \{1,\dots ,sl_{max}\}\}\right) \quad \forall i \in I \end{aligned}$$

8 $$\begin{aligned} \textit{atLeast}_{\lceil b_i^{min}/g \rceil }\left( \{U_{i,d,x} | d \in D, x \in \{1,\dots ,sl_{max}\}\}\right) \quad \forall i \in I \end{aligned}$$

*Maximum consecutive shifts* Each employee is only allowed to work for a maximum number of consecutive days before he must have a day off. This maximum limit is given to the problem as $c_{i}^{max}$ for each $i \in I$. In our formulation we state this constraint by introducing clauses that require a day off during all possible sequences of length $c_{i}^{max}$. Since this constraint assumes that the last day before the scheduling horizon sets a day off and the first day after the scheduling horizon also sets a day off, we do not need to consider any corner cases where a number of consecutive shifts lower than the maximum is scheduled directly next to the borders of the planning horizon.

9 $$\begin{aligned} \bigvee \limits _{x=0}^{c_i^{max}} X_{i,d+x} \quad \forall i \in I, d \in \{1,\dots ,|D|-c_{i}^{max}\} \end{aligned}$$

*Minimum consecutive shifts* Our problem requires that each employee works at least for a minimum number of consecutive days. In other words there is a minimum for the number of consecutive shifts, which is given as parameter $c_{i}^{min}$ for all $i \in I$, before an employee is allowed to have a day off.

Again we do not have to consider corner cases, since this constraint always assumes an infinite number of consecutive working days before and after the scheduling horizon. For all the other cases, we formulate this constraint by implicating the minimum length shift sequence whenever a new shift sequence starts after a day off:

10 $$\begin{aligned} \left( X_{i,d} \wedge \lnot X_{i,d+1}\right) \rightarrow \left( \bigwedge \limits _{x=2}^{c_i^{min}} \lnot X_{i,j+x}\right) \quad \forall i \in I, d \in \{1,\dots ,|D|-3\} \end{aligned}$$

*Minimum consecutive days off* This can be formulated similarly to the minimum consecutive shifts constraint. No corner cases have to be considered, as this constraint assumes an infinite sequence of days off before and after the scheduling horizon. The minimum limit of consecutive days off is given to the problem as parameter $o_i^{min}$ for each employee $i \in I$.

We again use a formulation variant which applies an implication of a minimum length day off sequence, similar as described for the minimum consecutive shifts constraint which we described previously:

11 $$\begin{aligned} \left( \lnot X_{i,d} \wedge X_{i,d+1}\right) \rightarrow \left( \bigwedge \limits _{x=2}^{o_i^{min}} X_{i,d+x}\right) \quad \forall i \in I, d \in \{1,\dots ,|D|-3\} \end{aligned}$$

*Maximum number of weekends* Whenever an employee has to work a shift on a Saturday or a Sunday in the schedule, the corresponding weekend is considered as a working weekend for this employee. The problem restricts the number of such working weekends for each employee *i* as parameter $a_i^{max}$. Because the scheduling always starts on Monday and ends on Sunday, the number of weekends can be easily calculated as $w={|D| \over 7}$. We can now introduce additional helper variables $W_{i,x}$ to state if an employee *i* works on the *x*-th weekend. We introduce the following equivalences to connect the *W* variables with the existing *X* variables in our formulation. Note that the *x* variables are multiplied with 7 in order to determine the day index of the *x*-th Sunday.

12 $$\begin{aligned} W_{i,x} \leftrightarrow (\lnot X_{i,(x \cdot 7)-1} \vee \lnot X_{i,x \cdot 7}) \quad \forall i \in I, x \in \{1,\dots ,w\} \end{aligned}$$

With the help of those variables we can then construct the following cardinality constraints to formulate the maximum number of weekends constraint:

13 $$\begin{aligned} \textit{atMost}_{a_i^{max}}(\{W_{i,1}, W_{i,2}, \dots , W_{i,w}\}) \quad \forall i \in I \end{aligned}$$

*Days off* An employee may have certain days on which it is strictly required that he has a day off. Those are given to the problem as sets of day indices $N_i$ for each employee *i*. We can introduce this in our formulation by simply generating the corresponding unit clauses:

14 $$\begin{aligned} X_{i,d} \quad \forall i \in I, d \in N_{i} \end{aligned}$$

### Modeling of soft constraints

In this section we give a description of all the soft constraints of the employee scheduling problem considered in this paper and specify how they can be encoded in our partial weighted maxSAT formulation.

*Requested shift types* Each employee may have some days where a certain shift type is requested for them to work. Since this is not a hard constraint, a violation will be penalized with a given weight. The corresponding penalties are given to the problem as parameters $q_{i,d,t}$, where $i \in I$, $d \in D$ and $t \in T$. We handle this constraint by inserting simple weighted unit clauses for all shift requests into our formulation:

15 $$\begin{aligned} S_{i,d,t} \cdot q_{i,d,t} \quad \forall (i,d,t) \quad where \quad \exists q_{i,d,t} \end{aligned}$$

*Unpreferred shift types* Similar to the requested shifts constraint, our problem may contain requests that require an employee to not work a particular shift on a certain day. Our formulation is again based on weighted unit clauses depending on problem parameters $p_{i,d,t}$ that set the weight of an unpreferred shift, where $i \in I$, $d \in D$ and $t \in T$:

16 $$\begin{aligned} \lnot S_{i,d,t} \cdot p_{i,d,t} \quad \forall (i,d,t) \quad where \quad \exists p_{i,d,t} \end{aligned}$$

*Cover requirements* A preferred number of employees that should be working in a shift type is defined for each day. This preferred value of working employees for shift *t* on day *d* is given to the problem in the form of parameters $u_{dt}$ for all $d \in D$ and $t \in T$. Furthermore for each of these values two penalty parameters $v_{dt}^{min}$ and $v_{dt}^{max}$ are used to penalize a possible under- or over-coverage of the preferred value.

We introduce two cardinality constraints per cover requirement to formulate this constraint. One for the over-coverage, which is penalized linearly depending on the weight $v_{dt}^{max}$, and the second one for the under-coverage also penalized linearly depending on the weight $v_{dt}^{min}$:

17 $$\begin{aligned} \textit{atMost}_{u_{dt}}(\{S_{1,d,t}, S_{2,d,t},\dots ,S_{|I|,d,t}\}) \cdot v_{dt}^{max} \quad \forall d \in D, t \in T \end{aligned}$$

18 $$\begin{aligned} \textit{atLeast}_{u_{dt}}(\{S_{1,d,t}; S_{2,d,t};\dots ;S_{|I|,d,t}\}) \cdot v_{dt}^{min} \quad \forall d \in D, t \in T \end{aligned}$$

### Using maxSAT to model nurse rostering

In this paper we provide a maxSAT formulation and computational results for the employee scheduling problem that was described by Curtois and Qu (2014). We chose this particular problem variant because it provides a number of challenging instances that are straightforward to use. However, the application of a partial weighted maxSAT formulation is not limited to this problem variant.

In this section we describe how the presented maxSAT formulation can be extended and adapted to model instances from the second international nurse rostering competition (INRC2) described by Ceschia et al. (2015). The problem considered for INRC2 asked for the assignment of nurses to shifts within a given planning horizon, subject to a number of hard and soft constraints. Note that INRC2 required its competitors to solve a sequence of consecutive weeks in the planning horizon in a multi-stage setting. We will not go into the details of this setting here and want to refer the interested reader to Ceschia et al. (2015) for more information.

In the following subsections we will briefly describe the constraints of the problem and also provide information on how they can be modeled using partial weighted maxSAT.

#### Additional variables for nurse rostering

The given nurse rostering problem has many similarities to the employee scheduling problem that we modeled in earlier sections of this paper, since the main goal of the problem is again to find the optimal roster for a number of given nurses and shift types. Therefore, in the following sections we will use the same decision variables and parameters that we have previously introduced if not noted otherwise. However, to model some of the constraints for the INRC2 instances we will also introduce some new variables and parameters which we describe now.

Since the given problem assigns a set of skills to each nurse, we define the set *K* as the set of all skills, and the sets $N^k, \forall k \in K$ which refer to the sets of all nurses that have a skill *k*. Furthermore, the sets $K^i, \forall i \in I$ contain all skills that are provided by a nurse *i*. Because a nurse can only use exactly one of its skills during a shift we also define the helper variables $U_{i,d,t,k}$ that are true if nurse *i* uses skill *k* in shift *t* on day *d*. The following hard constraints are then inserted to ensure that a nurse can only use one skill per shift:

19 $$\begin{aligned} \textit{atMost}_{1}\left( \left\{ U_{i,d,t,k} | k \in K^i \right\} \right) \quad \forall i \in I, d \in D, t \in T \end{aligned}$$

#### Hard constraints

This section will shortly present the hard constraints of the INRC2 instances and describe how they can be modeled using maxSAT.

*Single assignment per day* This constraint is the equivalent to the *An employee cannot be assigned more than one shift on a single day* constraint in Sect. 3.4 and can be modeled similarly.

*Under-staffing* The number of nurses for each shift and for each skill must be at least equal to the minimum requirement. The cover requirements can be expressed in the form of $c_{dtk}$, where $d \in D, t \in T, k \in K$ and the constraint can be modeled as follows:

20 $$\begin{aligned} \textit{atLeast}_{c_{dtk}}\left( \left\{ S_{i,d,t} \wedge U_{i,d,t,k} | i \in N^k \right\} \right) \quad \forall d \in D, t \in T, k \in K \end{aligned}$$

*Shift type successions* The shift type assignments of one nurse in two consecutive days must belong to the legal successions provided in the scenario. This constraint can be modeled like the similar constraint *disallowed shift sequences*, which is described in Sect. 3.4.

*Missing required skill* A shift of a given skill must necessarily be fulfilled by a nurse having that skill. This constraint is implicitly covered by the formulation for the *under-staffing* constraint.

#### Soft constraints

*Insufficient staffing for optimal coverage* This constraint is similar to *under-staffing* as it asks for an optimal number of nurses with a particular skill for each shift. It can be formulated similar to *under-staffing*, but as a soft cardinality constraint with a weighted penalty. The optimal cover requirements are given to the problem in a similar form as the cover requirements for *under-staffing*.

*Minimum consecutive working days* Assuming that the minimum number of consecutive working days is given as $c_{min}$, this constraint can be formulated as follows (since corner cases where a sequence of working days lies at a border of the planning horizon have to be considered as well, we define that any variable $X_{i,d}$ with a day *d*, that lies outside the scheduling horizon will always be set to true):

21 $$\begin{aligned} \begin{aligned}&\textit{atLeast}_{c^{min}}\left( \left\{ \lnot X_{i,d-1} \vee X_{i,d} \vee \left( \bigwedge \limits ^{j-1}_{k=0} \lnot X_{i,d+k} \right) | j \in \{1,\dots ,c_{min} \} \right\} \right) \cdot \textit{penalty}_{c^{min}} \\&\qquad \forall d \in D, i \in I \end{aligned} \end{aligned}$$

The idea behind this formulation is to insert a soft cardinality constraint for each position in the schedule. Whenever a new working day sequence starts on a position, the cardinality constraint will evaluate a set of clauses that count the length of the working day sequence, and will cause a penalty for each missing shift assignment if the length is lower than the minimum.

*Maximum consecutive working days* The maximum number of consecutive working days ($c^{max}$) can be formulated as follows (Similar to the *minimum consecutive working days* constraint we define that any variable $X_{i,d}$ that lies outside the schedule will be set to true):

22 $$\begin{aligned} \begin{aligned}&\textit{atMost}_{c^{max}}\left( \left\{ X_{i,d-1} \wedge \left( \bigwedge \limits ^{j-1}_{k=0} \lnot X_{i,d+k} \right) | j \in \{1,\dots ,|D|\} \right\} \right) \cdot \textit{penalty}_{c^{max}} \\&\qquad \forall d \in D, i \in I \end{aligned} \end{aligned}$$

Similar to the *minimum working days constraint* this formulation inserts cardinality constraints for each position in the schedule and counts the length of any working day sequence. A penalty will be caused for each shift assignment of a sequence that goes over the allowed maximum.

*Minimum/maximum consecutive working shifts* These constraints can be formulated similarly to the *minimum/maximum consecutive working days* constraints. However, because they ask for consecutive assignments of a certain shift type, we have to introduce new helper variables $O^t_{i,d}$ for each shift type *t* that will be true whenever employee *i* does not work in shift *t* on day *d*. Then we can model the minimum/maximum consecutive working shifts by just replacing the *X* variables with the corresponding *O* variables in the *minimum/maximum consecutive working days* constraints.

*Minimum/Maximum consecutive days off* These constraints can also be formulated similar to the *minimum/maximum consecutive working days constraints*, but with negated *X* variables. For example, the minimum number of consecutive days off constraint ($d_{min}$) can be formulated as follows:

23 $$\begin{aligned}&\textit{atLeast}_{d^{min}}\left( \left\{ X_{i,d-1} \vee \lnot X_{i,d} \vee \left( \bigwedge \limits ^{j-1}_{k=0} X_{i,d+k} \right) | j \in \{1,\dots ,d_{min}\}\right\} \right) \cdot \textit{penalty}_{d^{min}} \nonumber \\&\qquad \forall d \in D, i \in I \end{aligned}$$

*Preferences* This constraint will penalize any shift assignment which is undesired in respect to the nurse preferences. It can be formulated similar to the *Unpreferred shift types* constraint in Sect. 3.5.

*Complete week-ends* Nurses may ask to only work on complete week-ends. In this case they should either work on both week-end days or none. Any assigned weekend that is not complete will lead to a penalty. To model this constraint we define the set of all weekends as *W*, the set of all employees that have the complete weekend flag set to true as *CW* and define helper variables $W_{\textit{complete}_{i,x}}$, that are set to true if and only if weekend *x* is complete for employee *i*:

24 $$\begin{aligned} W_{complete_{i,x}} \leftrightarrow \left( \left( \lnot X_{i,(x \cdot 7)-1} \wedge \lnot X_{i,x \cdot 7}\right) \vee \left( X_{i,(x \cdot 7)-1} \wedge X_{i, x \cdot 7}\right) \right) \end{aligned}$$

25 $$\begin{aligned} \textit{atMost}_0\left( \{\lnot W_{complete_{i,x} | x \in W}\}\right) \cdot \textit{penalty}_{cw} \quad \forall i \in CW \end{aligned}$$

*Total assignments* For each nurse the total number of assignments must be included within a given minimum and maximum. This constraint can be formulated similar to the *maximum number of shifts* constraint in Sect. 3.4 but on a global level for all shift types and as a soft cardinality constraint with a weighted penalty.

*Total working week-ends* This constraint can be formulated similar to the *maximum number of weekends* constraint in Sect. 3.4, but as a soft cardinality constraint with a weighted penalty.

## Computational results

We now give an overview of our experimental environment and describe how our benchmark tests were executed and evaluated.

### Experimental environment

We conducted a large number of experiments with generated maxSAT encodings for the 24 instances that were described by Curtois and Qu (2014). The planning horizon of the instances ranges from two weeks to 52 weeks, while the number of considered employees ranges from 8 to 150. As this dataset contains very large instances it provides challenging benchmarks for solution techniques. If not noted otherwise we ran all of our experiments on an Intel Xeon E5345 2.33 GHz machine with a total of 48GB RAM. The encoded maxSAT instances are available online in DIMACS format and can be downloaded at.^1^

In our benchmarks we used two solvers which performed well on timetabling instances in the maxSAT evaluation 2015: WPM3 (Ansótegui et al. 2015) and Optiriss using the default configuration. The latter uses the riss framework (Kahlert et al. 2015) in combination with the publicly available OpenWBO solver (Martins et al. 2014). Both solvers were ranked first and second in the industrial category for partial weighted maxSAT problems. Besides being the leaders in their category, both solvers have also shown to provide good results for high school timetabling and timetabling instances, which share similarities with the considered employee scheduling problem.

### Comparison of different cardinality constraint encodings

Because our model utilizes a number of cardinality constraints, a crucial point in the configuration of our experiments turned out to be the determination of which cardinality constraint encodings we should use in order to get good results with the maxSAT solvers. There are five constraints which are affected in our formulation: The *cover requirement* constraint, the *workload requirement* constraint, the *maximum number of shifts* constraint, the *maximum number of weekends*, and the *One shift per day* constraint. For those, we applied four different encoding variants: *combinatorial encoding*, *sequential encoding*, *cardinality networks encoding*, and *bit adder encoding*. We used the implementation by Demirovic and Musliu (2014) to encode those constraints.

If we would consider all possible combinations for encoding the cardinality constraints in our model, we would have to generate and compare a total of $4^5 = 1024$ different formulations for each problem instance. In order to reduce this large amount of possibilities, we decided to investigate the number of generated variables and clauses for all constraint/encoding pairs in order to gather a first insight on their importance. We can see the results for one instance in Table 1.

**Table 1:** Overview on the number of generated variables (vars.) as well as the hard- and soft-clauses (h.c. and s.c.) for all the cardinality constraint/encoding pairs for instance 5

		Combinatorial	Sequential	Cardinality N.	Bit adders
Cover Req.	vars.	10,192	7616	7056	5096
	h.c.	35,616	28,672	21,168	17808
	s.c.	896	896	896	896
Workload Req.	vars.	Out of memory	6176	8032	5088
	h.c.	Out of memory	20,240	21,760	14864
	s.c.	Out of memory	0	0	0
Max shifts	vars.	Out of memory	3010	3520	6374
	h.c.	Out of memory	11,928	10,658	22,646
	s.c.	Out of memory	0	0	0
Max weekends	vars.	0	124	160	176
	h.c.	46	420	496	602
	s.c.	0	0	0	0
One shift per day	vars.	0	896	896	1344
	h.c.	448	2688	3136	4480
	s.c.	0	0	0	0

The combinatorial encoding turned out to be impractical in most cases and we were often not able to generate maxSAT encodings for many of the instances when using it. The huge amount of produced clauses required by this encoding forced our model generator to run out of memory when dealing with larger instances. When looking at the numbers displayed in Table 1, we can also see that the *maximum number of weekends* and the *one shift per day* constraints have a relatively low impact when compared with the other constraints. As this behavior appeared also in other instances, we decided to use only the sequential encoding for the *maximum number of weekends* constraint and only the combinatorial encoding for the *one shift per day* constraint in the remainder of our experiments. With the elimination of the combinatorial encoding in our configuration options because of the caused inconveniences with larger instances, and only three constraints remaining, we now have to examine only $3^3 = 27$ possible combinations.

In order to determine the best configuration for both WPM3 and Optiriss, we selected nine instances of different sizes and ran experiments with all the 27 possible encoding variants under a time limit of 30 min. The results of those experiments can be seen in Tables 2 and 3 for Optiriss and WPM3 respectively.

**Table 2:** Best results found by Optiriss using different combinations of cardinality encodings

Optiriss	Best solutions found in 30 min time limit				
Cardinality encoding	Inst. 2	Inst. 4	Inst. 7	Inst. 9	Inst. 11
seq./seq./seq.	837	5626	13,333	12,655	40,435
seq./seq./card.	837	5626	12,000	11,659	40,435
seq./seq./adder	839	5122	10,078	11,533	23,720
seq./card./seq.	840	6002	15,318	12,460	32,768
seq./card./card.	840	6002	12,111	12,242	32,768
seq./card./adder	838	5215	11,474	12,758	24,905
seq./adder/seq.	841	5407	14,319	11,044	34,612
seq./adder/card.	841	5407	15,148	11,662	34,612
seq./adder/adder	840	5331	11,785	12,752	25,633
card./seq./seq.	841	5609	13,813	14,353	32,281
card./seq./card.	841	5609	15,211	11,423	32,281
card./seq./adder	**834**	5723	11,987	13250	25,631
card./card./seq.	**834**	6210	14,080	12,156	37,028
card./card./card.	**834**	6210	13,779	13,154	37,028
card./card./adder	841	5316	10,682	10,641	22,130
card./adder/seq.	837	5711	13,002	12,570	32,618
card./adder/card.	837	5711	13,492	12,785	32,618
card./adder/adder	838	5504	9689	12,976	24,844
adder/seq./seq.	844	3900	5762	7729	15,916
adder/seq./card.	844	3900	5741	7526	15,916
adder/seq./adder	852	3720	5228	7437	16,624
adder/card./seq.	853	**3608**	5421	**6394**	**15420**
adder/card./card.	853	**3608**	5852	6804	**15,420**
adder/card./adder	847	3918	5452	7239	16,464
adder/adder/seq.	845	3907	5411	7716	16,627
adder/adder/card.	845	3907	5746	7422	16,627
adder/adder/adder	850	3798	**5040**	7215	16,436

Optiriss	Best solutions found in 30 min time limit				
Cardinality encoding	Inst. 12	Inst. 14	Inst. 16	Inst. 18	
seq./seq./seq.	57,680	17,959	15,584	35,073	
seq./seq./card.	58,369	16,665	15,584	39,555	
seq./seq./adder	34,964	17,549	13,263	25,829	
seq./card./seq.	57,575	16,761	15,635	37,084	
seq./card./card.	54,138	16,630	15,635	37,641	
seq./card./adder	33,939	18,362	14,544	23,932	
seq./adder/seq.	61,229	17,454	16,013	34,284	
seq./adder/card.	52,854	16,043	16,013	28,074	
seq./adder/adder	36,632	15,555	13,937	27,604	
card./seq./seq.	72,062	19,358	16,093	37,501	
card./seq./card.	49,699	18,247	16,093	38,147	
card./seq./adder	32,074	17,934	14,776	28,188	
card./card./seq.	56,279	19,044	16,903	37,778	
card./card./card.	50,404	15,546	16,903	35,638	
card./card./adder	32,239	16,918	14,880	26,855	
card./adder/seq.	62,154	18,980	17,419	38,269	
card./adder/card.	49,096	18,565	17,419	30,601	
card./adder/adder	33,340	18,593	15,990	29,781	
adder/seq./seq.	28,602	10,076	12,546	21,039	
adder/seq./card.	31,000	9875	12,546	22,548	
adder/seq./adder	28,694	**8777**	12,223	21,095	
adder/card./seq.	28,598	9776	13,026	20,710	
adder/card./card.	30,324	9144	13,026	**20,225**	
adder/card./adder	29,596	9555	13,049	20,280	
adder/adder/seq.	**27,193**	9758	11,939	20,462	
adder/adder/card.	29,606	9931	11,939	20,504	
adder/adder/adder	29,417	9756	**11,707**	20,996	

The first column describes the cardinality encodings used for the *cover requirement*/*workload requirement*/*maximum number of shifts* constraints. Encoding names have been abbreviated: seq. = sequential encoding, card. = cardinality networks, adder = bit adders. In each column the best result is formatted in boldface

**Table 3:** Best results found by WPM3 using different combinations of cardinality encodings

WPM3	Best solution found in 30 min time limit				
Cardinality encoding	Inst. 2	Inst. 4	Inst. 7	Inst. 9	Inst. 11
seq./seq./seq.	**828**	3189	5510	10,631	12,183
seq./seq./card.	**828**	3189	**4596**	10,949	12,183
seq./seq./adder	**828**	3494	8959	10,248	23,420
seq./card./seq.	**828**	3090	7446	11,132	**11,516**
seq./card./card.	**828**	3090	6545	11,405	**11,516**
seq./card./adder	**828**	**2688**	8351	12,154	24,114
seq./adder/seq.	**828**	2784	7712	12,178	12,478
seq./adder/card.	**828**	2784	8553	**10,033**	12,478
seq./adder/adder	**828**	2893	9364	10,964	24,195
card./seq./seq.	835	3394	5230	10,605	17,224
card./seq./card.	835	3394	6815	11,037	17,224
card./seq./adder	**828**	4082	7562	11,062	25,444
card./card./seq.	**828**	3087	7143	10,240	13,888
card./card./card.	**828**	3087	8147	10,942	13,888
card./card./adder	839	3704	9670	10,531	25,626
card./adder/seq.	840	3695	7543	11,871	15,393
card./adder/card.	840	3695	7760	11,235	15,393
card./adder/adder	**828**	3103	9287	12,374	22,719
adder/seq./seq.	1550	3718	10,502	13,982	29,673
adder/seq./card.	1550	3718	11,315	12,780	29,673
adder/seq./adder	1159	3198	9478	14,674	26,133
adder/card./seq.	1563	3994	9365	11,256	31,595
adder/card./card.	1563	3994	9253	12,773	31,595
adder/card./adder	856	4212	9791	12,693	25,827
adder/adder/seq.	1469	4108	10,292	10,771	29,083
adder/adder/card.	1469	4108	9476	11,963	29,083
adder/adder/adder	1359	3702	10,100	10,935	26,467

WPM3	Best solution found in 30 min time limit				
Cardinality encoding	Inst. 12	Inst. 14	Inst. 16	Inst. 18	
seq./seq./seq.	23,937	18,045	**10,292**	19,771	
seq./seq./card.	**18,770**	16303	**10,292**	18,498	
seq./seq./adder	1,697,590	15,297	12,738	21,408	
seq./card./seq.	22,010	15,419	12,528	19,191	
seq./card./card.	19,845	16,285	12,528	19,241	
seq./card./adder	1,697,590	16,654	16,099	22,100	
seq./adder/seq.	22,536	17,130	12,550	**17,277**	
seq./adder/card.	22,734	16,330	12,550	20,139	
seq./adder/adder	1,697,590	15,155	15,031	20,793	
card./seq./seq.	24,142	18,272	12,015	2,1095	
card./seq./card.	23,726	18,948	12,015	22,605	
card./seq./adder	32,150	18,455	14,126	29,910	
card./card./seq.	24,206	16,321	12,848	25,567	
card./card./card.	23,716	16,864	12,848	21,097	
card./card./adder	1,697,590	**14,915**	16,176	24,417	
card./adder/seq	25,331	17,055	13,360	24,620	
card./adder/card.	26,272	18,104	13,360	25,051	
card./adder/adder	1,697,590	17,490	16,998	30,144	
adder/seq./seq.	48,422	18,356	18,064	31,426	
adder/seq./card.	44,948	19,731	18,064	29,955	
adder/seq./adder	38,822	18,376	16,497	27,336	
adder/card./seq.	42,744	19,131	16,259	28,860	
adder/card./card.	44,272	18,959	16,259	31,694	
adder/card./adder	37,582	18,494	16,343	30,929	
adder/adder/seq.	42,648	15,723	17,590	31,791	
adder/adder/card.	45,583	20,184	17,590	27,403	
adder/adder/adder	35,857	18,143	18,593	29,081	

The first column describes the cardinality encodings used for the *cover requirement*/*workload requirement*/*maximum number of shifts* constraints. Encoding names have been abbreviated: seq. = sequential encoding, card. = cardinality networks, adder = bit adders. In each column the best result is formatted in boldface

A comparison of those results reveals that there is no general best combination of cardinality constraint encodings and good encodings are highly dependent on the solver which is used. While Optiriss prefers the adder encoding for the *cover requirements* constraint, the sequential encoding shows the best results for WPM3. We selected the best candidates for each solver by considering the sums of the results over all instances for each combination of cardinality encodings. The encodings which led to the minimum of all those sums were then taken to generate the instances for our final experiments. Therefore, the combinations of cardinality constraint encodings used for Optiriss were as follows: bit adder encoding for the *cover requirements* constraint, cardinality networks for the *workload requirements* constraint, and the sequential encoding for the *maximum number of shifts* constraint. The combinations of cardinality constraint encodings for WPM3 on the other hand were: The sequential encoding for the *cover requirements* constraint as well as the *workload requirements* constraint, and the encoding which uses cardinality networks for the *maximum number of shifts* constraint.

### Final experiments and comparison of solvers

By using the encodings mentioned above, we were able to create maxSAT instances for the original problems 1–21. Although our formulation can be used to encode Instances 22–24, unfortunately we could not generate maxSAT instances for those two problems, since our generator ran out of memory due to their large size (about 20 GB). Our final experiments were conducted using both solvers, giving them a time limit of 4 h for each of the 21 instances. The results of those benchmark tests can be seen in Table 4.

**Table 4:** The final results obtained for Instance 1–21 using WPM3 and Optiriss, with the application of the selected cardinality constraint encodings described in this paper

Instances				WPM3	Optiriss	Branch and Price	Gurobi
Inst.	Weeks	Employees	Shifts				
I 1	2	8	1	**607**	**607**	**607**	**607**
I 2	2	14	2	**828**	835	**828**	**828**
I 3	2	20	3	1009	3475	**1001**	**1001**
I 4	4	10	2	3102	3608	**1716**	**1716**
I 5	4	16	2	4037	3645	1160	**1143**
I 6	4	18	3	6150	6941	1952	**1950**
I 7	4	20	3	4596	5421	1058	**1056**
I 8	4	30	4	11,018	7617	1308	1323
I 9	4	36	4	10,949	6394	439	439
I 10	4	40	5	16,435	15,350	**4631**	**4631**
I 11	4	50	6	12,183	15,420	**3443**	**3443**
I 12	4	60	10	18,770	28,598	4046	**4040**
I 13	4	120	18	6,110,163	69,203	–	3109
I 14	6	32	4	16,303	9776	–	1280
I 15	6	45	6	30,833	16,506	–	4964
I 16	8	20	3	10,292	13,026	3323	3233
I 17	8	32	4	22,002	22,073	–	5851
I 18	12	22	3	18,498	14,433	–	4760
I 19	12	40	5	1,698,538	50,274	–	5420
I 20	26	50	6	5,519,316	147,325	–	–
I 21	26	100	8	14,715,064	–	–	–

Information about the size of each instance is included in the first four columns of the table, which display the number of weeks of the scheduling horizon, the number of employees, and the number of shift types. For comparison, the objective values of the best known solutions using the exact methods that were described by Curtois and Qu (2014) are also included. Results formatted in bold face denote proven optimal solutions

If we compare the outcomes for WPM3 and Optiriss we are not able to point out a clear winner which performs better over all the instances. While WPM3 performs significantly better on the smaller instances (Instances 1–7 and 11–12), it does not produce good solutions for the larger instances (Instances 8–10 and 13–21). Using Optiriss provides better results when it comes to solving the larger instances, except for the last two instances where the solver could not find any solution under 4 h.

Comparing our approach with another existing exact method based on integer programming, which was provided by Curtois and Qu (2014) (last two columns in the table) we can conclude that both maxSAT solvers could not find new unknown optimal results. However they could provide optimal solutions for instances 1 and 2. Running the maxSAT solvers for 4 h resulted in finding solutions for two of the instances which could not be solved by the integer programming approach within 1 h on a different environment.

Although currently the state of the art solvers that are based on integer programming produce better results for many of the considered instances, the results show that maxSAT as an exact method gives promising results for employee scheduling problems. As many maxSAT solvers are publicly available and their performance is consistently improving, this approach can be useful to find solutions for staff scheduling problems.

### Analyzing the influence of the under-coverage soft-constraint

To further investigate the problem we performed additional experiments by simplifying the instances. We omitted all soft constraints except under-coverage (Eq. 18). We wanted to investigate this constraint because it shows to have the highest weight in all instances, and as such contributed to the objective value significantly more than others.

Optiriss provides the option to experiment with the Linear maxSAT algorithm (Berre and Parrain 2010). The Linear algorithm is an iterative upper bounding algorithm in which the SAT solver is repeatedly called and in each call clauses are added which constrain the objective value to be less than in the previous iteration. Therefore, this process is only repeated until the SAT solver reports unsatisfiable, in which case the previously calculated solution is the optimal one. The Linear algorithm is invoked in Optiriss by supplying the parameter *-algorithm=1*. As this algorithm is appropriate to be used in this scenario, below we report experiments using it.

Every feasible solution for the simplified instances is a feasible solution for the original problem as well, as removing soft constraints does not impact feasibility. In Table 5 we provide the results obtained after running experiments for 1 h. We compare the performance of Optiriss with the Linear algorithm on the original and simplified instances (see column 1 and column 2 of Table 5). In the case of the simplified instances, we present the costs obtained after converting the solution to the original instance. We used the same cardinality constraint encoding as we did previously for Optiriss.

**Table 5:** The results obtained by running Optiriss with the Linear maxSAT algorithm for 1 h on simplified and original instances

Instance	Linear	Simplified-linear	Optiriss (Table 4)
Instance 1	**607**	620	**607**
Instance 2	847	858	835
Instance 3	1236	1050	3475
Instance 4	1859	1787	3608
Instance 5	2202	1534	3645
Instance 6	5763	2637	6941
Instance 7	6541	1625	5421
Instance 8	15,105	2894	7617
Instance 9	13,496	1991	6394
Instance 10	–	6649	15,350
Instance 11	–	6434	15,420
Instance 12	–	22,838	28,598
Instance 13	–	70,242	69,203
Instance 14	–	6634	9776
Instance 15	–	24,988	16,506
Instance 16	18,074	4867	13,026
Instance 17	–	14,315	22,073
Instance 18	18,498	13,143	14,433

For comparison purposes, we provide the corresponding solution for Optiriss from Table 4 and the best known solutions obtained by exact methods that were described by Curtois and Qu (2014). Optimal solutions are formatted in bold face

The results obtained in Table 5 are interesting for two reasons. Firstly, in most cases when a solution could be generated, the obtained results with the described technique with the simplified instances outperformed the previous maxSAT experiments even though less time has been allocated. Secondly, the simplification proved to be a very useful improvement for the Linear maxSAT algorithm. Instances 19–24 where not included in the table as no solution could be generated with either encoding technique using the Linear maxSAT algorithm.

These results indicate that the under-coverage constraint has a high influence on the objective value, at least for the Linear maxSAT algorithm. Because of this, leaving the solver all the time to focus on the under-coverage constraint showed to be valuable. Using this technique new unknown optimal results could not be found, but the results suggest the importance of the under-coverage constraint. Focusing only on the under-coverage constraint could also be applied to other solving techniques, like integer programming methods or local search.

## Conclusion

In this paper we have introduced, to the best of our knowledge, for the first time a partial weighted Boolean maximum satisfiability model for variants of the employee scheduling problem and the nurse rostering problem. We further generated maxSAT instances using four different cardinality encoding methods and compared the effects of these methods on two maxSAT solvers. Our results showed that there is a need to experimentally select an efficient combination of cardinality encodings for each solver separately, as the best encoding strategy in our experiments varied depending on the used solver. A comparison between the two solvers could not point out a clear winner for all of the considered benchmark tests. While WPM3 performed better on smaller instances, Optiriss was able to produce better results for many of the larger instances.

Currently an exact approach based on integer programming provides better results than maxSAT for most of the considered instances. However, maxSAT could provide optimal solutions for two of the instances and obtained solutions for two very large instances within 4 h, which could not be solved by integer programming within 1 h.

Although the results for many instances are currently not competitive when compared with results produced by state of the art solvers that are based on integer programming, we have shown that maxSAT was successfully used to solve the majority of the problem instances. We think that the main reason why the results currently cannot compete with integer programming lies in the intensive use of cardinality constraints that are required to model the problem. The high significance of choosing good encoding strategies for cardinality constraints that became apparent during our experiments as well as the discovered influence of the under-coverage constraint indicate the importance of efficient strategies to deal with these types of constraints. We therefore conclude that there is a need for improving the performance of maxSAT solvers, especially regarding cardinality constraints. Seeing many maxSAT solvers becoming available and consistently advancing in the recent years, we believe that the provided instances for employee scheduling, which are now also part of the annual maxSAT evaluation, can serve as relevant benchmarks to evaluate new strategies for an efficient handling of cardinality constraints.

Possible improvements and extensions could be subject of future work when working with the proposed model. It would be interesting to investigate if we can break symmetries in our model. Further, given the findings regarding the under-coverage constraint, developing a lexicographic optimization approach for Employee Scheduling might be valuable. In this approach, one would first optimize for the under-coverage constraint and then optimize the rest of the soft constraints. Furthermore, a hybridization of maxSAT with heuristic techniques within the framework of very large neighbourhood search could be considered.

## Footnotes

1 http://www.dbai.tuwien.ac.at/research/project/arte/maxsat_employeescheduling/.

## References

[CR1] Ansótegui, C., Didier, F., & Gabàs, J. (2015) Exploiting the structure of unsatisfiable cores in maxsat. In *Proceedings of the twenty-fourth international joint conference on artificial intelligence, IJCAI 2015*, Buenos Aires, Argentina, July 25–31, 2015 (pp. 283–289).

[CR2] Asín, R., Nieuwenhuis, R., Oliveras, A., & Rodríguez-Carbonell, E. (2009). Cardinality networks and their applications. In *Proceedings of the 12th international conference on theory and applications of satisfiability testing, SAT 2009*, Swansea, UK, June 30–July 3, 2009 (pp. 167–180).

[CR3] Berre DL, Parrain A (2010). The SAT4J library, release 2.2. JSAT.

[CR4] Biere A, Heule M, van Maaren H, Walsh T (2009). Handbook of satisfiability, frontiers in artificial intelligence and applications.

[CR5] Bofill, M., Garcia, M., Suy, J., & Villaret, M. (2015). MaxSAT-based scheduling of B2B meetings. In *Proceedings of the 12th international conference integration of AI and OR techniques in constraint programming, CPAIOR 2015*, Barcelona, Spain, May 18–22, 2015 (pp. 65–73).

[CR6] Burke EK, Curtois T (2014). New approaches to nurse rostering benchmark instances. European Journal of Operational Research.

[CR7] Burke EK, Curtois T, Post GF, Qu R, Veltman B (2008). A hybrid heuristic ordering and variable neighbourhood search for the nurse rostering problem. European Journal of Operational Research.

[CR8] Ceschia, S., Thanh, N. D. T., Causmaecker, P. D., Haspeslagh, S., & Schaerf, A. (2015). Second international nurse rostering competition (INRC-II)—Problem description and rules. CoRR, arxiv.org/abs/1501.04177

[CR9] Chuin Lau H (1996). On the complexity of manpower shift scheduling. Computers & Operations Research.

[CR10] Curtois, T., & Qu, R. (2014). Computational results on new staff scheduling benchmark instances. Technical report, ASAP Research Group, School of Computer Science, University of Nottingham, NG8 1BB, Nottingham, UK

[CR11] Demirovic, E., & Musliu, N. (2014). Modeling high school timetabling as partial weighted maxSAT. Technical report, Technical University Vienna.

[CR12] den Bergh JV, Beliën J, Bruecker PD, Demeulemeester E, Boeck LD (2013). Personnel scheduling: A literature review. European Journal of Operational Research.

[CR13] Ernst AT, Jiang H, Krishnamoorthy M, Sier D (2004). Staff scheduling and rostering: A review of applications, methods and models. European Journal of Operational Research.

[CR14] Haspeslagh, S., Messelis, T., Berghe, G. V., & Causmaecker, P. D. (2013). An efficient translation scheme for representing nurse rostering problems as satisfiability problems. In: *Proceedings of the 5th International Conference on Agents and Artificial Intelligence, ICAART 2013*, Barcelona, Spain, 15–18 February, 2013 (Vol. 2, pp. 303–310).

[CR15] Haspeslagh S, DeCausmaecker P, Schaerf A, Stølevik M (2014). The first international nurse rostering competition 2010. Annals of Operations Research.

[CR16] Kahlert, L., Krüger, F., Manthey, N., & Stephan, A. (2015). Riss solver framework v5. 05. SAT-Race

[CR17] Kundu, S., & Acharyya, S. (2008). A SAT approach for solving the nurse scheduling problem. In *TENCON 2008—2008 IEEE region 10 conference, IEEE* (pp. 1–6).

[CR18] Martins, R., Manquinho, V. M., & Lynce, I. (2014). Open-WBO: A modular maxsat solver. In *Proceedings of the 17th international conference on theory and applications of satisfiability testing, SAT 2014, held as part of the Vienna Summer of Logic, VSL 2014*, Vienna, Austria, July 14–17, 2014 (pp. 438–445).

[CR19] Mohr, R., & Masini, G. (1988). Good old discrete relaxation. In *ECAI* (pp. 651–656).

[CR20] Römer, M., & Mellouli, T. (2016). Future demand uncertainty in personnel scheduling: Investigating deterministic lookahead policies using optimization and simulation. In *Proceedings of the 30th European conference on modelling and simulation, ECMS 2016*, Regensburg, Germany, May 31–June 3, 2016 (pp. 502–507). 10.7148/2016-0502.

[CR21] Sinz, C. (2005). Towards an optimal CNF encoding of Boolean cardinality constraints. In *Proceedings of the 11th international conference on principles and practice of constraint programming, CP 2005*, Sitges, Spain, October 1–5, 2005 (pp. 827–831).

